# Quantitative phenotyping of crop roots with spectral electrical impedance tomography: a rhizotron study with optimized measurement design

**DOI:** 10.1186/s13007-024-01247-7

**Published:** 2024-08-02

**Authors:** Valentin Michels, Chunwei Chou, Maximilian Weigand, Yuxin Wu, Andreas Kemna

**Affiliations:** 1 Geophysics Section, Institute of Geosciences, University of Bonn, Meckenheimer Allee 176, Bonn, 53115 NRW Germany; 2 Earth and Environmental Sciences Area, Lawrence Berkeley National Laboratory, 1 Cyclotron Road, Berkeley, 94720 CA USA

**Keywords:** Beans, Complex resistivity, Induced polarization, Maize, Phenotyping, Rhizotron, Root biomass, Root surface area, Spectral electrical impedance tomography

## Abstract

**Background:**

Root systems are key contributors to plant health, resilience, and, ultimately, yield of agricultural crops. To optimize plant performance, phenotyping trials are conducted to breed plants with diverse root traits. However, traditional analysis methods are often labour-intensive and invasive to the root system, therefore limiting high-throughput phenotyping. Spectral electrical impedance tomography (sEIT) could help as a non-invasive and cost-efficient alternative to optical root analysis, potentially providing 2D or 3D spatio-temporal information on root development and activity. Although impedance measurements have been shown to be sensitive to root biomass, nutrient status, and diurnal activity, only few attempts have been made to employ tomographic algorithms to recover spatially resolved information on root systems. In this study, we aim to establish relationships between tomographic electrical polarization signatures and root traits of different fine root systems (maize, pinto bean, black bean, and soy bean) under hydroponic conditions.

**Results:**

Our results show that, with the use of an optimized data acquisition scheme, sEIT is capable of providing spatially resolved information on root biomass and root surface area for all investigated root systems. We found strong correlations between the total polarization strength and the root biomass ($R^2 = 0.82$) and root surface area ($R^2 = 0.8$). Our findings suggest that the captured polarization signature is dominated by cell-scale polarization processes. Additionally, we demonstrate that the resolution characteristics of the measurement scheme can have a significant impact on the tomographic reconstruction of root traits.

**Conclusion:**

Our findings showcase that sEIT is a promising tool for the tomographic reconstruction of root traits in high-throughput root phenotyping trials and should be evaluated as a substitute for traditional, often time-consuming, root characterization methods.

**Supplementary Information:**

The online version contains supplementary material available at 10.1186/s13007-024-01247-7.

## Background

In order to meet the steadily rising demand for food from an increasing population, and react to the simultaneously progressing climate change, the resilience and yield of crops have to be improved in the near future [1]. Therefore, a significant amount of research is dedicated to breeding improved plant systems through phenotyping, conducted in both controlled laboratory conditions and field trials (e.g. [2]). Historically, advancements in trait phenotyping of above-ground parts of plant systems have progressed much more rapidly than the capability to characterize root systems, which therefore have been termed ‘the hidden half’ of plant systems [3]. Still, roots form the interface of plants to the soil, which makes understanding and observing them crucial for understanding the soil-plant continuum.

Traditionally, the retrieval of crop root traits, for example via root coring (e.g. [4]), trenching (e.g. [5]), the use of mini-rhizotron tubes (e.g. [6]) or shovelomics (e.g. [7]) and the subsequent analysis over scanning software, is labour-intensive and often highly invasive to the plant or soil around it. However, driven by progress in sensor technology and data analysis pipelines (e.g. [8]), efforts have been made to explore several non-invasive and high-throughput methods for root phenotyping. Especially in controlled laboratory conditions, image-based rapid phenotyping methods were developed to evaluate the root system structure (e.g. [9, 10]). Additionally, other technologies, such as magnetic resonance imaging (e.g. [11]), X-ray computer tomography (e.g. [12]) or neutron radiography (e.g. [13]), have been successfully used to image root growth in soil columns and rhizotrons. Although these methods are promising for laboratory studies and deliver well-resolved reconstructions of the root system, their applicability is often limited to small-scale experiments and, more importantly, they are not easily transferable to field root phenotyping applications.

Due to their cost-efficiency, non-invasiveness, and potential to sense the electrical properties of root systems, geoelectrical methods are increasingly being used for imaging and characterization of plant root systems and their surrounding environment (for an overview, see [14–16]). Electrical resistance measurements were used to identify high and low resistance zones within maize root segments (e.g. [17]), as well as to quantify the root surface area, number of lateral roots, and root length of root systems in hydroponic solution (e.g. [18]). An extension to this method is the electrical resistivity tomography (ERT), which allows imaging of the two- or three-dimensional resistivity distribution of a medium using numerical inversion schemes (e.g. [19]). ERT has been used to quantify the biomass of tree roots (e.g. [20, 21]), finer root systems (e.g. [22]), and carrots [23], although its ability to directly estimate root traits is limited by the necessary high resistivity contrast between root and soil (e.g. [16]). It is therefore more frequently employed as a tool to phenotype root systems indirectly, using changes in resistivity as a proxy for root water uptake dynamics of crops and trees (e.g. [24–28]). Some studies injected the electric current into the stem of the plant instead of the surrounding soil (a method known as Mise-à-la-masse) and utilized the source current density to locate where current is exiting the root, therefore providing information about the dimensions and architecture of the root system [29–31]. However, while [29] could successfully identify likely root water uptake zones, [30] and [32] reported problems with leakage currents exiting the plant stem area, preventing the interpretation of the data in terms of the root system’s extent.

In comparison to resistivity-based methods, capacitance and impedance measurements are able to additionally quantify the electrical polarizability of a medium, i.e., its ability to form electrical polarization (local charge separation and thus electrical energy storage) upon an imposed electric field. The first observations of polarization in plant roots using capacitance measurements have been reported by [33] and [34], and a large number of studies have since shown correlations of the capacitance with various root traits for single-frequency measurements (e.g. [35–42]) as well as for multi-frequency spectral measurements (e.g. [43]). More recently, [44] could link diurnal changes in the capacitance to the observed transpiration (and thus activity) of various plant types, and [45] showed an inhibition of this behaviour under Cadmium overexposure. Electrical impedance spectroscopy (EIS) measurements quantify the polarization effect over the phase shift between injected current and the resulting voltage signal, typically conducted at frequencies ranging from mHz to tens of kHz. These measurements have been employed to establish relationships between polarizability and root parameters such as root biomass and root surface area (e.g. [46, 47]) or root length (e.g. [48]). EIS is usually conducted by placing electrodes in the stem of the plant (e.g. [48]), within the medium embedding the plant roots (e.g. [47]), or on individual root segments (e.g. [49]).

Similarly to ERT, spectral electrical impedance tomography (sEIT) uses numerical inversion schemes to combine multiple EIS measurements into 2D or 3D images of complex resistivity or conductivity, thus enabling spatially resolved tomographic investigations. [50] used sEIT to image the spatial extent of an oilseed root system in hydroponics and showed changes in the electrical polarization response due to physiological root decay. Later research by [51] demonstrated that root system polarizability fluctuates under exposure to diurnal light cycles, suggesting a link between the electrical signature and the plant’s ion uptake processes. While EIS measurements have linked polarization strength to root biomass in barley and wheat [46, 47, 52], sEIT studies have so far focused on the qualitative spatial reconstruction of root systems [50, 51], rather than quantifying specific root traits from the tomographic polarization signature. A challenge in the quantitative interpretation of sEIT results is the spatially varying resolution capability in the imaging region, mainly determined by the geometry of the electrode array and the sensitivity patterns of the measurement configurations. In geophysics, many studies attempted to improve the measurement design in geoelectrical surveys using optimization algorithms (e.g. [53–57]). However, to our knowledge, the optimization procedure was never performed for laboratory tank geometries.

In this study, we aim to address the so far lacking quantitative evaluation of sEIT measurements in the context of root phenotyping and propose a laboratory workflow to extract spatially resolved root traits from the obtained tomographic polarization signature. To maximize reconstruction capabilities of the method in a rhizotron, we used an optimization algorithm to generate an improved data acquisition scheme and assessed its performance for the reconstruction of root traits. We performed sEIT measurements on maize, pinto bean, black bean, and soy bean plants in hydroponics and established an empirical relationship between the imaged polarization strength in the root system and validation root traits recovered from optical scanning.

## Material and methods

### Electrical polarization of root systems

In general, electrical polarization processes take place in regions with concentration gradients formed between charged surfaces and the electroneutral pore or cell solution. These regions are referred to as the electrical double layer (EDL), and are comprised of a layer of adsorbed ions near the charged surface (Stern layer), and a diffusive layer reaching into the fluid (e.g. [58]). While in soils and rocks the EDL is present at the interface between mineral grain surface and pore water, in root systems, it also forms within cell membranes, apoplastic and symplastic pathways [59], as well as on the outer root surface (e.g. [36]). During current injection, electromigrative forces disturb the charge structure of the EDL in an electric field, which leads to a relaxation towards electroneutrality after this external field is shut off. This displacement effect is known as induced polarization and manifests itself as a time delay between the excitation current and the measured voltage signal, or as a phase shift between measured voltage and induced current for frequency-domain measurements (e.g. [19]).

The resulting polarization signature of the root is influenced by three major factors. The first factor is the EDL strength, which is controlled by the surface charge of root surface and cell walls and the ionic composition of the inter- and outer-cellular electrolyte (e.g. [60]). Therefore, structural characteristics of the root system, for example root surface area (e.g. [41, 47]), root volume (e.g. [61]), or root biomass (e.g. [40, 47]) can be derived from both capacitance and impedance measurements. Furthermore, ion uptake processes of the root temporarily alter the charge structure of the EDL, and thus magnitude of polarization. Consequently, time-series polarization signatures can provide insights into the physiological activity of plants, such as their response to day-night cycles [44, 51] or physiological decay [45, 50].

The second factor controlling the polarization is the point of current injection and voltage measurement. Injecting the current directly into root segments [49] or into the stem of the plant [48] generally leads to large phase shifts of up to 500 mrad, while current injection into the surrounding medium [47, 50] results in significantly smaller phase shifts of only a few mrad. This phenomenon can be explained by the measurement’s sensitivity to the root polarization. Injecting current into the stem or root segment forces the current through the internal root structure, primarily capturing the “intrinsic” response of the root and stem. In contrast, injecting into the surrounding medium allows the current to flow around the root system, resulting in a mixed contribution from soil, water, and root. Although this approach weakens the overall polarization magnitude by bypassing the highly polarizable root, it enables a tomographic evaluation of the root system (e.g. [50, 51]).

The final factor influencing the resulting polarization signature is the length scale of polarizable structures in the root system. Larger length scales (e.g., larger cells) lead to longer relaxation times ($\tau$), the period ions need to return to an equilibrium state. This relaxation time can be used to infer the characteristic length scales within a medium (e.g., [62–65]). For example, in granular porous media, the relaxation time can be linked to the particle radius (e.g. [58, 66, 67]) with

1 $$\begin{aligned} \tau = \frac{r^2}{2D}, \end{aligned}$$

where *r* is the particle radius and *D* the diffusion coefficient of ions in the Stern layer. Since the relaxation time is inversely related to the measurement frequency, multi-frequency impedance and capacitance measurements can be used to identify dominant polarization length scales in the root system. Using Eq. 1, [50, 51] suggested that root polarization in their experiments occurred on the $\mu$m scale, indicating (at least partially) cell-scale polarization processes. This is supported by the observation that in all studies using EIS or sEIT, polarization strength increases towards higher frequencies in the kHz-range (e.g., [47, 49–51, 61, 68, 69]), corresponding to smaller polarization length scales. Contrarily, [47] suggested that the outer root surface might be the main polarization source in EIS/sEIT measurements when the current electrodes are not placed within the stem, indicating polarization scales in the higher $\mu$m- to lower mm-range. [70] later proposed a model showing that during injection outside of the root system, polarization occurs primarily on the outer root surface, while stem injection polarizes the entire cell wall surface area within the root system, resulting in stronger polarization signatures and shorter relaxation times. These contrasting findings show that the question about the scale of polarization within root systems is still not fully answered, and further research is needed to better understand its underlying processes.

### Spectral electrical impedance tomography

The sEIT method uses a number of four-point electrode configurations to measure the impedance of a medium. In each individual measurement, two of the electrodes are used to inject an alternating current $I^*(\omega )$ with angular frequency $\omega$ into the investigated medium, while the other two electrodes are used to record the potential difference (voltage) $U^*(\omega )$ between them. The ratio of potential difference to injected current is the complex-valued, frequency-dependent impedance $Z^*(\omega )$:

2 $$\begin{aligned} Z^* = \frac{U^*}{I^*} = Z' + iZ'', \end{aligned}$$

with $Z'$ being the real part and $Z''$ the imaginary part of the impedance, and *i* the imaginary unit ($i^2=-1$). Alternatively, $Z^*$ can be written in polar notation:

3 $$\begin{aligned} Z^* = |Z^*| e^{i\varphi _{\textrm{Z}}}, \end{aligned}$$

where $|Z^*|$ is the impedance magnitude and $\varphi _{\textrm{Z}}$ the phase angle between current and voltage signals. The impedance can be converted to a so-called apparent complex resistivity $\rho _a^*$ via a (real-valued) geometric factor *K* that is depending on the arrangement of electrodes in the measurement:

4 $$\begin{aligned} \rho _a^* = KZ^*. \end{aligned}$$

Several spatially differing impedance measurements are combined to derive a 2D or 3D image of the complex conductivity distribution using tomographic inversion algorithms (e.g. [19]). Similar to the impedance, the complex conductivity $\sigma ^*$ (or its inverse, the complex resistivity $\rho ^*$) can be split up into a real and imaginary part, or magnitude and phase as

5 $$\begin{aligned} \sigma ^* = \frac{1}{\rho ^*} = \sigma ' + i\sigma '', \qquad |\rho ^*| e^{i\varphi _{\rho }}. \end{aligned}$$

Here, the real part $\sigma '$ describes the conduction, in-phase properties, whereas the imaginary part $\sigma ''$ describes the polarization, out-of-phase properties of the medium.

In this study, we use the finite-element based complex conductivity inversion code CRTomo [71], which uses a non-linear iterative Gauss-Newton scheme to compute the distribution of $\sigma ^*$ over a range of measurement frequencies, where each frequency is inverted separately. Since the inversion problem is ill-posed, first-order model smoothing is used to regularize the inversion process. The numerical calculations were performed on a two-dimensional, triangular grid with refined cell size near the electrodes to ensure a high computational accuracy. The data misfit in the inversion process is weighted by error estimates for the impedance magnitude and phase values, giving more importance to measurements that are assigned with a smaller error estimate. Analogously to [72], impedance magnitude errors $\Delta |Z^*|$ were assumed to follow a linear error model:

6 $$\begin{aligned} \Delta |Z^*| = a |Z^*| + b, \end{aligned}$$

with *a* being the relative and *b* the absolute impedance magnitude error. For the phase error $\Delta \varphi _{\textrm{Z}}$, a constant phase error model [71] was assumed:

7 $$\begin{aligned} \Delta \varphi _{\textrm{Z}} = c. \end{aligned}$$

Note that other models for phase error estimation exist, for example [73], who propose an inverse power-law relationship between phase error and resistance—however, this method could not be applied due to missing normal-reciprocal measurements in this study.

### Optimized measurement design

In electrical imaging, the choice of measurement scheme (i.e., set of four-point electrode configurations) can significantly impact quality and interpretability of the dataset. Each configuration has a unique, spatially varying sensitivity to changes in the subsurface resistivity distribution. Consequently, low-sensitivity areas impede accurate model parameter reconstruction in the inversion—in our study, ultimately leading to an underestimation of root properties. While the geophysical community established standardized measurement schemes for geoelectrical field surveys (e.g., Wenner, Schlumberger, dipole-dipole, or multiple gradient arrays [19]), they are not easily applicable to a closed laboratory tank geometry due to varying tank layouts, tank dimensions, or electrode arrangements. Studies in the past usually relied on customized dipole-dipole or Wenner-type configurations for data acquisition (e.g. [74] for ERT and [73] for sEIT measurements). Although this is an intuitive approach, it is not ideal because sensitivity patterns within a closed geometry are more complex than in surface electrode spreads, and areas in the rhizotron could be underrepresented by the chosen measurement configurations.

**Fig. 1:**
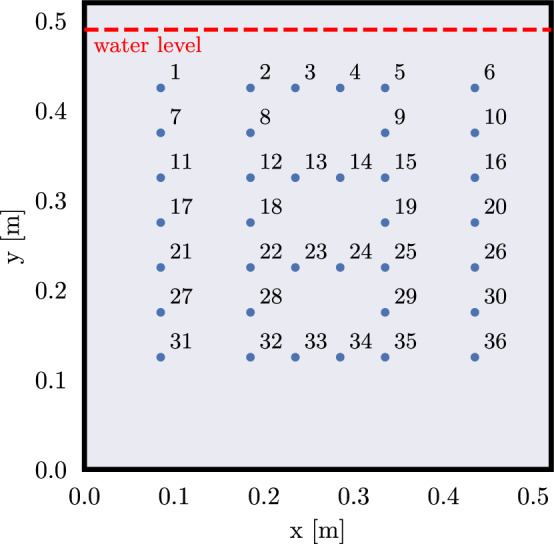
Sketch of the electrode layout in the rhizotron. The dashed red line indicates the water level during the experiments. A set of 36 electrodes, indicated as blue dots, was used for data acquisition

Therefore, in this study, we optimize the measurement scheme with the “Compare-R” approach described in [75]: Beginning with a sparse starting scheme, a set of measurement configurations is created by an iterative algorithm that searches for configurations whose sensitivity distributions maximize the diagonal entries of the so-called model resolution matrix. This matrix describes how well the model parameters, recovered from the data, fit the true model parameters—for further information on this topic, the reader is referred to [76]. We implemented the code in Python using the PyGimli framework [77] to calculate the matrix containing the sensitivities of each configuration (also referred to as the Jacobian matrix). Although we are conducting complex resistivity inversions in this study, the optimization was only done for the real part of the complex resistivity. This simplification is motivated by the fact that we do not expect strong polarization signatures, as past experiments with root systems have only shown small phase values of up to approximately -30 mrad [50, 51]. Therefore, the imaginary part of the complex sensitivity pattern is expected to be very similar in shape to the real part [78], making it unnecessary to include both in the algorithm. The optimization was focused on the expected rooting zone (*x*=0.085$-$0.435 m and *y*=0.125$-$0.425 m in Fig. 1) using spatial weighting factors, as implemented in [55]. Additionally, we limited the maximum number of current injections for the measurement scheme to 40 dipoles in order to stay in a reasonable data acquisition time window of approximately one hour (for measurement frequencies ranging from 0.1 Hz to 45 kHz). In total, the final scheme consists of 800 measurement configurations.

To highlight the importance of homogeneous resolution characteristics for the objective of this study, we conducted a synthetic experiment comparing the computed optimized set with a reduced set of measurements that excluded configurations utilizing the central batch of electrodes within the rhizotron. For the reduced set, this exclusion is introducing a low-sensitivity zone in the centre of the rhizotron. An artificial polarization signature, mimicking a root system with a phase shift of -25 mrad, was created, and synthetic datasets were computed for both the full and reduced sets of measurements. We generated 10 synthetic datasets for each measurement scheme, contaminating each with Gaussian noise ensembles (1% relative and 0.001 $\Omega$ absolute impedance magnitude errors, and 0.5 mrad absolute phase error) using unique seeds to randomize noise contributions. All datasets were then inverted using CRTomo, with error estimates matching the assumed noise distribution errors. To mitigate the effects of data outliers from specific noise realizations, we averaged the results of all 10 inversions for the optimized and reduced sets. The reconstruction capability of both schemes was quantified using the Pearson correlation coefficient (PCC) and structural similarity index (SSIM), as for example used in [75]. These parameters can vary between -1 and 1, where a value of 1 indicates a perfect positive correlation between two datasets [79].

### Spectral analysis

To analyze the spectral behaviour of the measurement data, we used the Debye decomposition scheme as implemented in [80]. The complex resistivity distributions obtained from the inversions were described by this empirical model using a defined number of Debye relaxation terms (one for each relaxation time in a predefined range depending on the measurement frequencies):

8 $$\begin{aligned} \rho ^*(\omega ) = \rho _0 \left( 1-\sum _{k=1}^{N} m_k \left[ 1-\frac{1}{1+i\omega \tau _k}\right] \right) \end{aligned}$$

Here, $\rho _0$ denotes the (real-valued) direct-current resistivity, $m_k$ the *k*th chargeability, and $\tau _k$ the *k*th relaxation time for the *k*th Debye relaxation term. From the retrieved relaxation time distribution, so-called integral parameters can be computed that describe the overall polarization behaviour of the investigated medium. In this study, the most relevant parameters retrieved are

- the total chargeability $m_{\textrm{tot}} = \sum _{k=1}^{N}m_k$ as a measure of the total polarization strength,
- and the mean logarithmic relaxation time $\tau _{\textrm{mean}} = \textrm{exp}(\sum _{k=1}^{N}m_k \textrm{log}(\tau _k)/\sum _{k=1}^{N}m_k)$ as an indicator for the average timescale of polarization processes within the system.

A more detailed description of these parameters can be found in [80].

### Experimental setup

In preparation for the experiment, maize (*Zea mays*), soy bean (*Glycine max*), black bean and pinto bean (*Phaseolus vulgaris*) were germinated and grown in paper bags filled with gardening soil. A growth lamp was used to accelerate plant growth and emulate a natural day-night-cycle. Because plant root systems can develop differently when grown under hydroponic conditions [81], the choice was made to grow the plants in soil to create a root architecture that is more similar to that of an in-situ root system.

To conduct the sEIT measurements, the same rhizotron setup as described in [30] was used. It has inner dimensions of $52\hbox {cm}\times 52\hbox {cm}\times 2.5\hbox {cm}$ and a clear front and back window to allow the visual inspection of the plant root system inside. The rhizotron has slots for 64 electrodes, but since the measurement device was limited to 36 channels, only this number of silver/silver chloride (Ag/AgCl) wire electrodes were used for the setup, protruding 0.5 cm into the inner rhizotron. The layout of the electrodes is shown in Fig. 1.

For all of the measurements, tap water with a conductivity of $$105.1 \pm 2.9\,\hbox {$\mu$S/cm}$$ and temperature of $$21.8 \pm 0.5\,^\circ \hbox {C}$$ (given in mean and standard deviation) was used as a background medium. Because the temperature does not show strong variations, we do not expect significant effects of temperature on the impedance measurement signal, as for example shown in [82]. We chose to use water as a background medium, because it does not exhibit significant polarization responses, therefore leaving only the polarization of the root system to be measured (e.g. [50]). The water level was kept at a height of 3 cm below the upper opening of the rhizotron to ensure a constant upper boundary of the modelling domain during the inversion process of the datasets. Other recorded environmental parameters are the air temperature and relative humidity in the lab, which respectively ranged from 20 to $$30\,^\circ \hbox {C}$$ and 15 to 50 % over the whole span of the experiments.

### sEIT data acquisition and processing

**Fig. 2:**
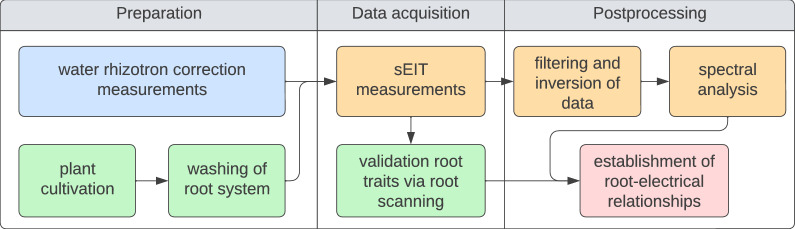
Methodology of the experiment, divided in preparation stage (correction measurements, plant cultivation and root system washing), data acquisition stage (sEIT measurements and collection of validation root traits), and postprocessing stage (filtering and inversion of data, spectral analyis, and comparison of root and electrical data)

The workflow of the sEIT experiments is showcased in Fig. 2. Previously to each sEIT measurement, the whole plant was removed from the soil container and washed with tap water. After the root system was deemed to be clear of soil particles, the plant was placed into the rhizotron and fixed in position with a piece of tape. Following the sEIT measurement, the plant was removed from the rhizotron, and a new plant was placed into the rhizotron for the next sEIT survey. Sometimes, tiny root segments that ripped off the main root system were still floating in the rhizotron after plant removal—however, since the amount of biomass was negligible in comparison to the whole root system, we do not expect that this had a strong influence on the following measurements. To achieve a wider range of root system variability, we conducted measurements on plants in different growth stages. An overview of all measured plants and their respective age at the time of data collection (day after sowing, DAS) is shown in Table 1. Overall, measurements on 7 maize plants, 7 black bean plants, 5 pinto bean plants and 4 soy bean plants within the age range of 13 to 73 days after sowing were conducted. The sEIT datasets were acquired at 40 frequencies in the range from 0.79 Hz to 45 kHz using the EIT40 impedance tomograph developed by [83], which took approximately one hour per plant system. The device is optimized for multichannel usage by measuring the potential at all remaining electrodes for each current injection. Through superposition of the transfer impedances of the resulting three-point measurements, any four-point configuration for a specific injection electrode pair can be computed.

**Table 1:** Overview of the number of collected datasets per plant age range (day after sowing)

Plant type	DAS			
	0-20	21-40	41-60	61-80
maize	3	2	2	0
black bean	1	3	3	0
pinto bean	2	1	1	1
soy bean	0	2	1	1

The raw data was corrected to account for the 2D inversion of a dataset that was collected in a 3D domain [50], as well as for polarization effects caused by the measurement setup. For this, sEIT measurements on only water were performed prior to some of the plant measurements. The procedures for both corrections are described in Supplementary Material 1.

After processing of the raw data, inversions were performed for all frequencies between 0.79 Hz and 1 kHz. We chose to not invert higher-frequency data because of limits in phase measurement accuracy of the used device [83]. For the impedance magnitude errors, a relative error of $a=2\%$ and absolute error of $b = 0.01$ $\Omega$ (according to 1‰ of the lowest encountered values in the measurements) were assumed for all inversions. Because we anticipate slightly larger phase measurement errors for higher frequencies, the constant phase error was set to half of the standard deviation of the phase values for a given frequency. For all inversions, we made sure that the error-weighted root-mean-square (RMS) error between predicted and recorded data was close to 1 (e.g. [71]), indicating that the actual data are well described by the predicted data within the assumed error estimates.

As a last step, a Debye decomposition over the frequency range of 0.79 Hz to 1 kHz was performed for all complex resistivity values obtained from the inversions, resulting in a set of integral polarization parameters, i.e. chargeability and relaxation time, for each cell in the grid.

### Comparison of root and electrical parameters

During the experiment, root validation data were collected for each investigated plant (Fig. 2). For this, the root systems were segmented into vertical sections of 10 cm height after the electrical measurements, and subsequently scanned with an LA2400 scanner by Regent Instruments. The root images were analyzed with the root image analysis software WinRHIZO Pro 2017a by Regent Instruments in order to retrieve the total root surface area and average root diameter for each section within the rhizotron. After scanning, the roots were weighed, dried in a low-temperature oven for 72 h and weighed again to obtain the root wet and dry biomasses.

We compared the recovered root traits with the chargeabilities and relaxation times extracted from all inversion grid cells that lie within the root area. Here, the root area refers to the area that we classified as “containing roots” using the photos of the rhizotron front. Based on the hypothesis that a higher amount of root matter leads to larger retrieved chargeabilities, we compare the average total chargeability ($\bar{m}$) in a root zone with the corresponding validation root traits (root biomass $M_{\text {bio}}$ and root surface area $A_{\text {surf}}$). From the underlying polarization mechanisms and results of previous studies (e.g. [47]), we expect that the average chargeability is proportional to the root trait density in the root zone volume:

9 $$\begin{aligned} \bar{m} \sim \frac{M_{\text {bio}}}{V_{\text {rz}}} \sim \frac{A_{\text {surf}}}{V_{\text {rz}}}, \end{aligned}$$

where $V_{\text {rz}}$ is the volume of the root zone. Considering the different cell volumes of the used (irregular) grid, the average chargeability is given as

10 $$\begin{aligned} \bar{m} = \frac{\sum _{k=1}^{n} V_k m_{\text {tot},k}}{\sum _{k=1}^{n} V_k}, \qquad \sum _{k=1}^{n} V_k = V_{\textrm{rz}}, \end{aligned}$$

with *n* being the number of grid cells within the root zone, $m_{\text {tot},k}$ the chargeability of the *k*th cell, and $V_k$ the volume of the *k*th cell. From Eq. 9 and 10, we see that the sum of the chargeabilities times their corresponding cell volume is proportional to the desired root traits. The averaged chargeability integrated over the root zone ($m_{\text {rz}}$) is then given as

11 $$\begin{aligned} m_{\text {rz}} = V_{\textrm{rz}}\bar{m} \sim M_{\text {bio}} \sim A_{\text {surf}}. \end{aligned}$$

A similar approach is introduced for the mean relaxation time of each cell. Following Eq. 1, we want to test the hypothesis that [70] proposed in their mechanistic model, stating that the main polarization length scale in sEIT measurements corresponds to the root diameter. We are comparing the averaged mean relaxation time with the average root diameter in a root zone. For the averaged mean relaxation time in the root zone ($\bar{\tau }$), we obtain

12 $$\begin{aligned} \bar{\tau } = \frac{1}{V_{\textrm{rz}}}\sum _{k=1}^{n} V_k\tau _{\text {mean}, k} \sim \bar{d_{\text {r}}}^2, \end{aligned}$$

where $\tau _{\text {mean}, k}$ is the mean relaxation time of the *k*th cell, and $\bar{d_{\text {r}}}$ the average root diameter in the given root volume $V_{\text {rz}}$.

## Results

### Effect of optimized measurement design

**Fig. 3:**
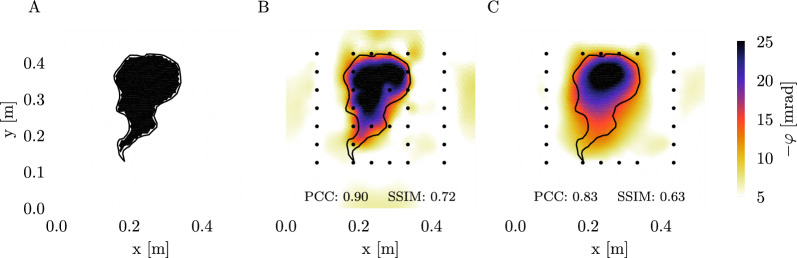
True resistivity phase model (**A**) and inversion results for the optimized (**B**) and reduced (**C**) measurement schemes. The black dots indicate electrode positions, the black line delineates the shape of the phase anomaly in the true model

In the following, we present the results of the synthetic study described in the "Optimized measurement design" section. The inversion results in Fig. 3 show that the optimized scheme is able to resolve the true root zone, indicated as a black outline, well in both shape and magnitude (with a PCC of 0.9 and a SSIM of 0.72). There is a sharp contrast between the reconstructed phase anomaly and background, and only towards the edges of the anomaly, there is a slightly reduced phase shift in comparison to the true model. On the other hand, the missing electrodes, and therefore reduced sensitivity coverage in the second scheme, lead to a worse reconstruction of the anomaly (with a PCC of 0.83 and SSIM of 0.63). Especially the lower part of the anomaly is weakly resolved, and the contrast between anomaly and background is not as strong as for the optimized scheme.

### Data quality

**Fig. 4:**
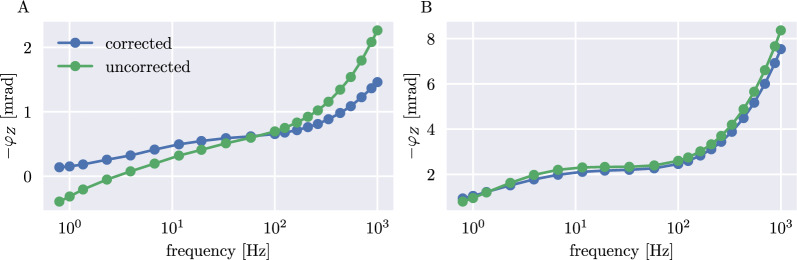
Corrected and uncorrected impedance phase spectra of black bean plant BB_2 (**A**) and pinto bean plant PB_5 (**B**). Note that the negative impedance phase shift -$\varphi _{\text {Z}}$ is plotted

The data quality of an sEIT survey can be assessed by evaluating the smoothness of the recorded spectra, the contact impedance of the electrodes, as well as the magnitude of current that is being lost by electrical leakage paths towards system ground [83]. For all datasets, we did not observe sudden jumps in the impedance spectra that would indicate faulty measurements—in Fig. 4, exemplary resistivity phase spectra of a single four-point configuration (electrodes 3, 10, 13, 32) of two root systems at different growth stages (BB_2 at 21 DAS (A) and PB_5 at 63 DAS (B)) are shown. Here, we also want to highlight the influence of the phase correction method described in the "sEIT data acquisition and processing" section. While the correction strength varied from configuration to configuration, overall, the procedure led to less positive phase values in the lower-frequency range and reduced phase shifts at higher frequencies. Additionally, it is evident that for bigger plants with stronger polarization signatures, the correction is less important because of the overall larger signal. The contact resistances for all measured quadrupoles were in the range of $10-40\,\textrm{k}\Omega$, and the ratio of injected to leakage current between $10^3$ and $10^5$, indicating an overall good data quality. The only data points that were disregarded resulted from a malfunctioning electrode amplifier that was recognized too late for the first five measured plants, leaving only 718 of the 800 configurations usable.

### Root system imaging

**Fig. 5:**
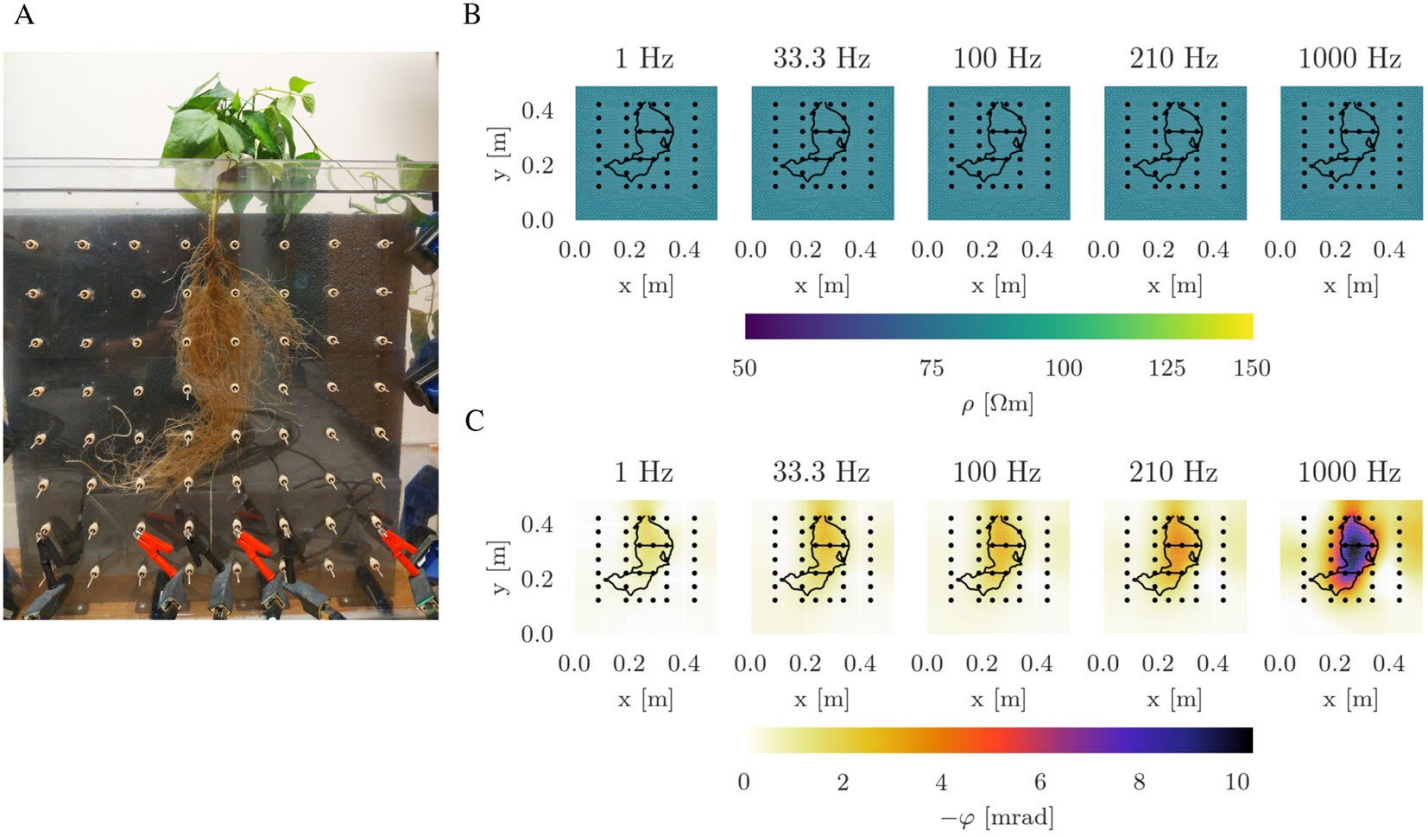
**A** Exemplary pinto bean plant (PB_5), embedded in water rhizotron. **B** Complex resistivity magnitude image at five frequencies, obtained from the inversion. **C** Complex resistivity phase shift at five frequencies, obtained from the inversion. The extent of the root system is shown as black outlines in the inversion results

**Fig. 6:**
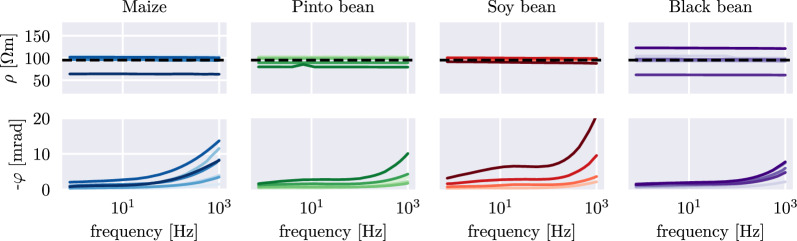
Exemplary complex resistivity magnitude and phase spectra for maize, pinto bean, soy bean and black bean, extracted from grid cells within the root zone (approximately 5–10 cm below the connection point between stem and roots) for all measured plants. Different shades of colour mark data from different root systems. The horizontal dashed black line indicates the mean resistivity of the water within the rhizotron

In the tomographic inversion results, we first investigated the spatial reconstruction ability of sEIT with regard to the extent of the root system, shown exemplary for pinto bean plant PB_5 in Fig. 5. The retrieved resistivity magnitude image does not show significant variations between background and rooting area, and therefore aligns with the results of previous hydroponic tomographic studies [50, 51]. This is the case for all investigated plants—an overview of the resistivity magnitude images at 1 kHz is shown in Fig. 12. Only when the roots were densely packed in the rhizotron (e.g. SB_3 and SB_4 due to a large root system), a slight resistivity increase can be observed in the rooting area. In contrast to the resistivity magnitude, the recovered phase image reveals a clear polarization response in the root zone, which varies in strength from plant to plant (for all plants, see Fig. 13). In areas where no roots are present, the phase shifts are consistently close to zero, regardless of measurement frequency. In common for all phase imaging results of the root zone is a frequency dependence of the polarization strength, manifesting in only slight phase shifts in the low-frequency (1 Hz) range and increasingly stronger phase shifts up to -25 mrad at 1 kHz. This pattern can be observed in the results for all investigated plants (Fig. 6). While the spectra near the stem and central part of the root system tend to have stronger phase shifts, the reconstructed spectra of the outer regions of the root zones still differ significantly from these of the surrounding water background. Except for a few slight peaks in the lower-frequency range (for example soy bean at around 10 Hz, see Fig. 6), most of the phase spectra extracted from the root zone do not exhibit pronounced peaks.

### Spectral analysis results

**Fig. 7:**
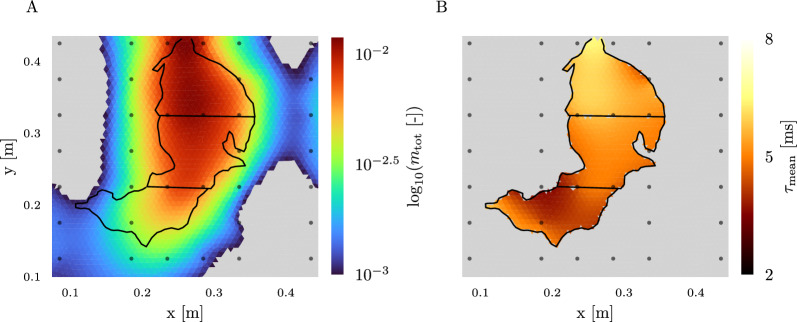
Total chargeability **A** and mean relaxation time** B** for the root area of one of the investigated pinto bean plants. A chargeability below $10^{-3}$ is considered as background and therefore masked in grey, the root area is delineated by black lines. For the mean relaxation time, we masked cells outside of the root system area, as values in this area are uncertain due to the low to zero polarizability in this region

We performed the spectral analysis of the complex resistivity spectra for each cell of the inversion grid using the Debye model described in the "Spectral analysis" section. From this, we obtained the spatial distribution of the total chargeability and mean relaxation time for each plant root system. In Fig. 7, the distribution of these parameters is displayed for one of the pinto bean plants (PB_5). Similar results were obtained for the other plant systems (see Fig. 14). The highest chargeability values are observed in the upper part of the root system close to the stem area, corresponding to the stronger phase shifts in this region. While the centre part of the root system shows an equally strong, or only slightly reduced polarization strength, the lower part of the root system gradually exhibits lower chargeabilities. This is in line with the results reported by [50, 51]. The relaxation time distributions do not follow clear spatial patterns. While some show, similar to the chargeability, higher relaxation times in the upper region of the root system (for example the pinto bean plant in Fig. 7), others exhibit an almost uniform distribution over the whole root system area or even higher relaxation times in the lower region of the rooting area. No correlation of the pattern with the stem or root region, type of plant, or age is found. For a complete overview of all relaxation time distributions, see Fig. 15.

**Fig. 8:**
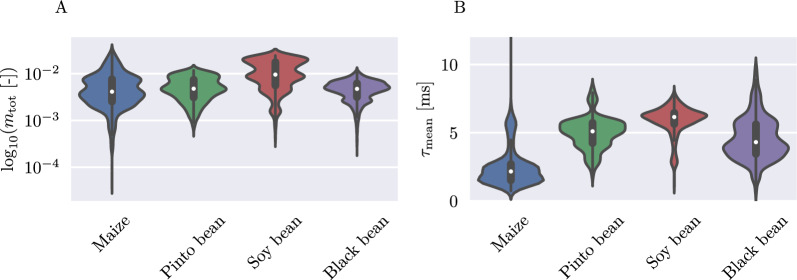
Violinplots showing the distribution of total chargeability** A** and mean relaxation time** B** within the rooting area for all plant types. White dots indicate the mean of each distribution

Comparing the retrieved chargeability distributions of the root zones for each plant type, we found that all plant types show similar polarization strengths with mean total chargeabilities between $10^{-3}$ and $10^{-2}$ (Fig. 8A). Overall, the soy bean plants show the highest polarization strength. The mean relaxation times (Fig. 8B) for all plant types lie within 1 and 12 ms, with maize showing the lowest mean relaxation times (1–2 ms).

### Comparison of electrical measurements with root validation data

**Fig. 9:**
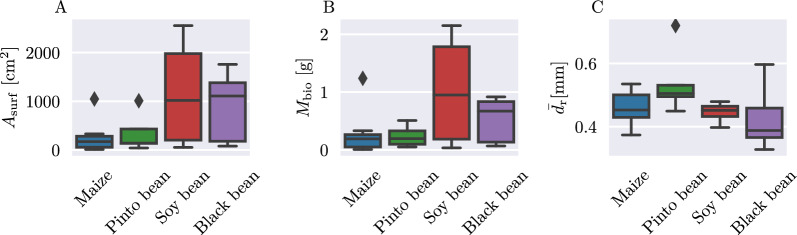
Overview of retrieved root parameters for each plant type. Displayed are the distributions of the total root surface area (**A**), total root biomass (**B**) and average root diameter (**C**)

An overview of the validation root parameters is given in Fig. 9. Overall, the total root biomass and root surface area of the maize plants are the lowest, while soy bean has the highest measured root surface area and biomass for a single plant with $2554\,\text {cm}^2$ and 2.151 g, respectively. The average root diameter is in a similar range of 0.35 to 0.55 mm for all plant types, with black bean having the lowest and pinto bean the highest mean values.

**Fig. 10:**
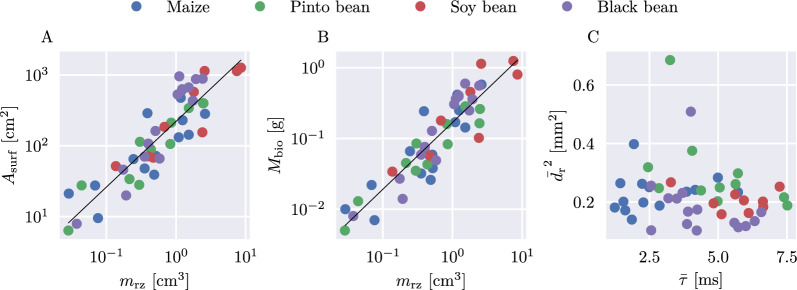
**A** Total root surface area plotted against integrated root-zone chargeability ($m_{\text {rz}}$). **B** Total dry root biomass plotted against $m_{\text {rz}}$. **C** Square of the average root diameter, plotted against the averaged mean relaxation time

We found a strong correlation between the integrated root-zone chargeability $m_{\text {rz}}$ and the total root biomass and total root surface area, respectively (Fig. 10). The established relations for both root parameters are

13 $$\begin{aligned} A_{\text {surf}} \approx 221 \times m_{\text {rz}}^{0.93}, \end{aligned}$$

14 $$\begin{aligned} M_{\text {bio}} \approx 0.169 \times m_{\text {rz}}^{0.94}, \end{aligned}$$

where $A_{\text {surf}}$ is the total root surface area in $\text {cm}^2$, $M_{\text {bio}}$ the dry root biomass in g and $m_{\text {rz}}$ the integrated root-zone chargeability in $\text {cm}^3$. With a coefficient of determination of $R^2 = 0.8$ and PCC of 0.89 for the root surface area and $R^2 = 0.82$ and PCC of 0.91 for the root biomass, the goodness of these power-law fits is comparable to the relationships reported in [47] for four-point EIS measurements. It is notable that all four plant types express a similar relationship between root biomass, root surface area and integrated chargeability. Furthermore, the exponents in Eq. 13 and 14 (i.e., the slope in Fig. 10) are similar, and close to one. Therefore, for small values of the integrated chargeability ($m_{\textrm{rz}} \le 2$), one can assume a linear behaviour between $m_{\textrm{rz}}$ and both $A_{\text {surf}}$ and $M_{\text {bio}}$, respectively.

The averaged mean relaxation time $\bar{\tau }$ of the root zone does not correlate with any of the collected root parameters, including the square of the average root diameter according to Eq. 1 (Fig. 10).

## Discussion

### Application of optimized measurement schemes

In this study, the application of an optimized measurement scheme led to an improved reconstruction of root traits. The simulation results indicate that achieving a homogeneous coverage of measurement configurations is essential for fully reconstructing a polarization anomaly within the rhizotron. The findings emphasize that the experimental setup of EIS and sEIT surveys can lead to significant variations in the sensitivity distribution, and therefore resolution of the investigated target. Considering the tomographic reconstruction capability of a measurement setup is therefore crucial for accurately estimating root traits and ensuring comparability of results across different studies. However, it is worth noting that the influence of measurement sensitivity is different for the intrinsic polarization parameters derived from the Debye decomposition.  [84] showed that, while the recovered chargeability exhibits a strong dependence on the resolution characteristics, the relaxation time is less affected and can also be decently reconstructed at lower sensitivities. Therefore, if the relaxation time is in the focus of the study, a lower-resolution measurement setup might be sufficient for acceptable results.

Additionally, the optimization procedure in this study was performed for ERT surveys and used for an sEIT survey under the assumption that the low-polarizable background leads to similar sensitivity characteristics. For applications with stronger polarizability contrasts, the optimization should be performed with the consideration of complex sensitivities, as cross-sensitivities between real and imaginary parts of data and model become non-negligible (e.g., [85, 86]).

### Reconstruction quality of rooting area and root parameters

The resistivity phase images and inferred chargeability images for all plants (Fig. 13 and Fig. 14) show a consistently well reconstructed root zone polarization anomaly. Overall, these results are in agreement with the results obtained by [50, 51], although we believe that the outer dimensions of the root systems are even sharper resolved in this study due to the application of an optimized measurement scheme. Note that in the previous studies, frequencies above 300 Hz were disregarded, and the better delineation of the polarization anomaly might be partly due to the incorporation of higher measurement frequencies of up to 1 kHz. This is also supported by the findings of [61], who retrieved better correlations between capacitance and root volume for higher measurement frequencies (10 kHz and higher). It is notable that for some of the maize plants (M_5, M_6 and M_7), the lower part of the root system expresses a low-chargeability zone. This effect is most likely a result of the root preparation process prior to the measurements. Especially for the larger maize root systems, the fine roots were difficult to clean, resulting in longer washing times and consequently higher loss of root biomass (see Fig. 9). As evident in Fig. 10, the reduced root matter in these areas also led to a decreased recovered chargeability. For future experiments with similar methodology, it might be advisable to use a growth medium that is easier to clean off the roots, for example sand, plant granulate, or soil with low clay content.

The relationships expressed in Eq. 13 and 14 allow a spatially resolved reconstruction of root parameters, depending solely on the chargeability distribution recovered from the electrical measurements. The similar exponents in both power-law expressions are likely caused by a linear correlation between root biomass and root surface area (e.g. [87]). Here, we want to stress that chargeabilities may vary when the Debye decomposition is performed with data from other frequency ranges, and the resulting relationships may differ in the calibration parameter values from those presented here. Especially for fine root systems, where polarization peaks in the 10–20 kHz range are reported (e.g., [49]), we expect that if only frequencies up to 1 kHz are considered in the spectral analysis, as done in this study, the total chargeability may be underestimated. Still, our results should be transferable to other studies when measuring in a similar frequency range and, therefore, could be used without prior knowledge (i.e. photos) of the root zone. When other frequency ranges are used, the calibration parameter values have to be adjusted to account for the characteristic polarization response in the specific frequency range.

**Fig. 11:**
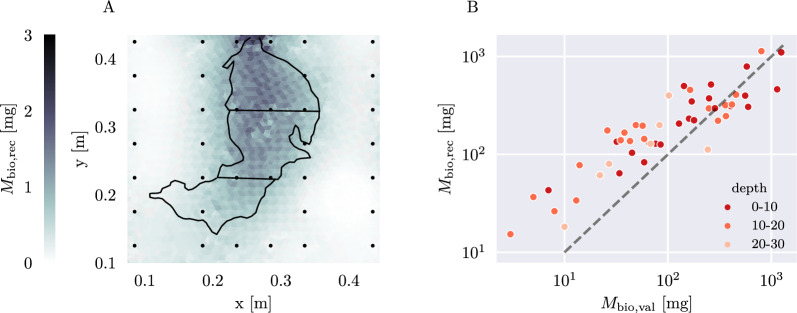
**A** Reconstructed biomass per cell for pinto bean plant PB_5. The black outline marks the rooting area of the plant, black dots denote the electrode positions.** B** Reconstructed root biomass for each root zone ($M_{\text {bio, rec}}$), compared to the validation root biomass estimated by scanning ($M_{\text {bio, val}}$). The three different root zone depths within the rhizotron (in cm) are displayed in shades of red, the perfect match of reconstructed and validation biomass is indicated as a dashed grey line

**Fig. 12:**
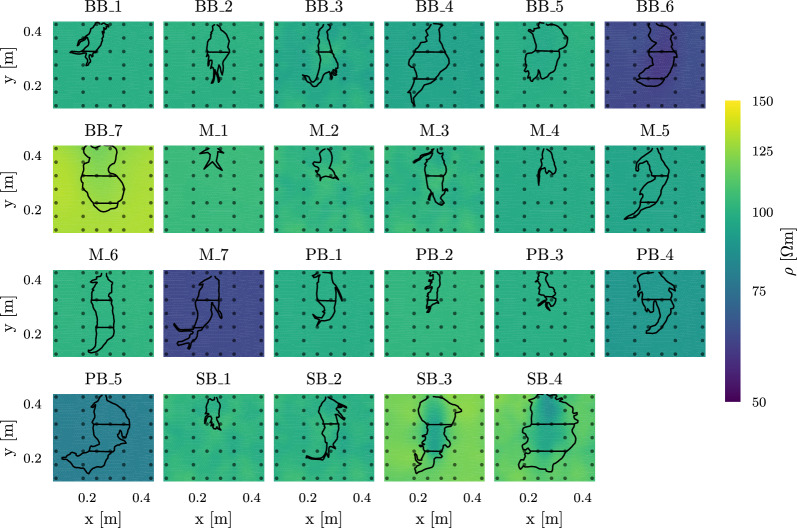
Complex resistivity magnitude images at 1 kHz for all measured plants. The plot labels indicate black bean (BB), maize (M), pinto bean (PB) and soy bean (SB) plants

**Fig. 13:**
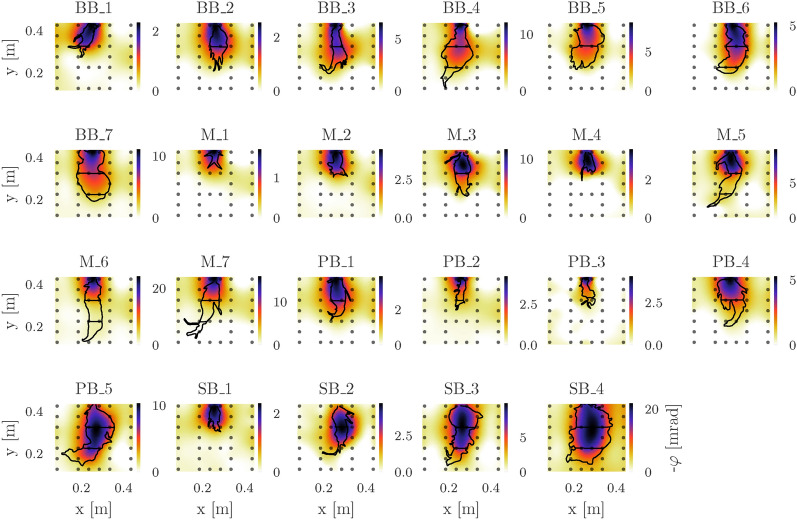
Complex resistivity phase images at 1 kHz for all measured plants. The plot labels indicate black bean (BB), maize (M), pinto bean (PB) and soy bean (SB) plants. Note that for better visibility, images are plotted with different colourbars

**Fig. 14:**
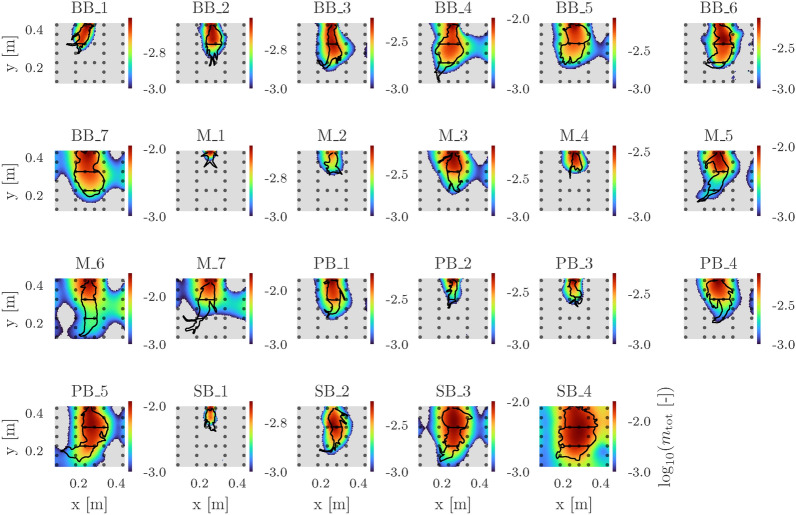
Total chargeability images for all measured plants. The plot labels indicate black bean (BB), maize (M), pinto bean (PB) and soy bean (SB) plants. Masked, gray areas indicate total chargeabilities below $10^{-3}$. Note that for better visibility, images are plotted with different colourbars, and colourbar values are logarithmic

**Fig. 15:**
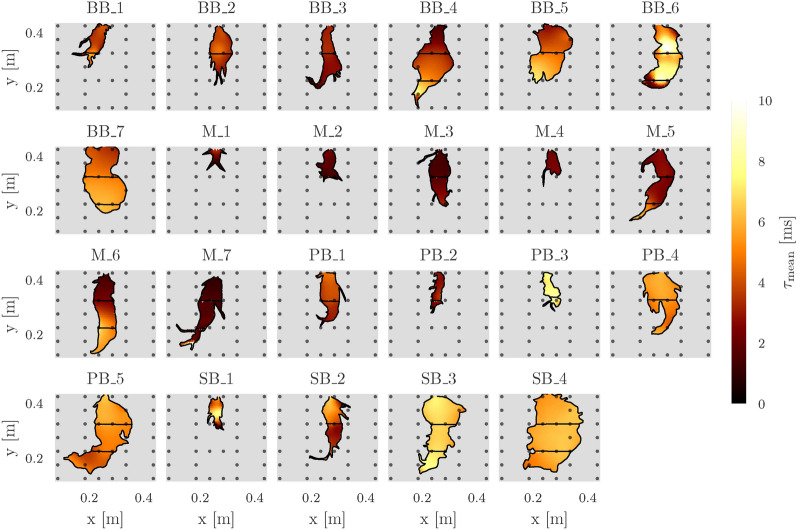
Mean relaxation time images for all measured plants. The plot labels indicate black bean (BB), maize (M), pinto bean (PB) and soy bean (SB) plants. Areas outside of the rooting area are masked in gray

In Fig. 11A, the biomass distribution for pinto bean plant PB_5, derived from its chargeability, is displayed. While delivering a spatially resolved biomass image, the discretization with different cell sizes in the grid introduces non-smooth transitions between cells. Additionally, the biomass outside of the root area close to the boundary between root zone and non-root zone is overestimated. Comparing the reconstructed biomass ($M_{\text {bio, rec}}$), obtained from the chargeability distribution, with the validation biomass ($M_{\text {bio, val}}$) estimated by scanning for all plants (Fig. 11B), one can see a good agreement between both datasets, especially for higher biomass values. However, in root areas with lower ($\le 100\,\hbox {mg}$) root biomass, the chargeability-derived biomass slightly overestimates the true biomass. These areas are most often encountered in the lower region of the plant root system (depths 10–20 cm and 20–30 cm). Both this effect and the effect of the biomass overestimation outside of the root zone is most likely caused by the smoothing constraint in the inversion.

### Variability between plant types

The observed relationship between the root biomass (and root surface area) and the integrated chargeability (Fig. 10) reveals a similar trend for all four investigated plant types. Although maize is a monocot with a fibrous, fine root system and therefore has a different root anatomy than bean plants (dicots) that have a thicker, central tap root with fine lateral roots [88], no significant difference in the obtained chargeability images can be observed. This result supports the hypothesis that the polarization strength in this experiment is mainly controlled by the total polarizable surface area within the root system, i.e. the surface area of the inner cell membranes—as this surface area is unrelated to the macroscopic architectural traits of monocot and dicot root systems, no differences in chargeability between both root types can be expected. However, root architecture could play a significant role when a large resistivity contrast between rooting medium and root exists, as the root matter could act as a conductive network with anisotropic electrical properties. More research is needed to investigate the influence of different root architectures in a high-resistivity rooting medium, and in this case, current injection into the stem might be advantageous to force current flow through the root, for example as applied in the MALM method by [30]. Instead of low-frequency (pseudo direct-current) injection, it could be promising to utilize-high frequency ($> 10\,\hbox {kHz}$) injection currents to primarily capture the cell membrane polarization (e.g. [61]), possibly also reducing problems with leakage currents encountered in previous studies.

### Length scale of polarization

The length scale of polarization, expressed as relaxation time in the measurement data, is believed to correspond to the internal and external plant root morphology (e.g. [60]). However, we do not see a clear dependence of the mean relaxation time with the root diameter, as suggested by [70]. We explain this observation in two ways: Firstly, as the root diameter is varying between 0.3 and 0.5 mm (or the squared root diamter between 0.1 and $0.25\,\text {mm}^2$), the range of observed diameters might not be large enough to imply a measurable variation of the relaxation time. For future experiments investigating the polarization length scale, care should be taken to select plant types that show a wide variability in root diameters. Secondly, the polarization response might not be dominated by charge separation on the root diameter scale, but by cell-scale polarization processes, as mentioned already in the discussion of the chargeability. This would explain the overall narrow range of mean relaxation times. Considering typical diffusion coefficient values of $10^{-9}\,\text {m}^2$/s (adopted from [51]), relaxation times in the ms range indicate polarization at the $\mu \hbox {m}$ scale (Eq. 1), likely related to internal root structures such as inner cell walls, or the distance between Casparian strips and endodermis bridged by apoplastic and symplastic pathways [51]. Using a frequency range of 0.79 Hz to 1 kHz, according to Eq. 1, our measurements are sensitive to a scale of polarization between 0.56 and $20.07\,\mu \hbox {m}$, which is significantly smaller than the average observed root diameter of approximately 0.5 mm. To capture polarization processes on this scale, a measurement frequency of 1.3 mHz, resulting in a relaxation time of approximately 125 s, would be needed. We advise using lower measurement frequencies in future experiments if macroscopic polarization length scales above 0.1 mm are of interest. Additionally, measurements of the average cell size within the root segments could be taken and linked to high-frequency relaxation times.

### Application in phenotyping experiments

Our results highlight that sEIT is a useful tool for rapid phenotyping of a variety of fine crop root systems in hydroponic conditions. In combination with automated inversion and spectral analysis, reducing the number of measurement frequencies or number of current injections, or increasing the number of simultaneously measured plants, could still significantly lower data acquisition and processing time. Not only is the method able to estimate the root traits of a plant, but it can also localize high- and low-density root zones and delineate the outer shape of the root system. Additionally, although not shown in this study, another important aspect of spectroscopic impedance methods is its applicability towards physiological processes in the plant [51, 61], which is information that can not be recovered by image-based phenotyping methods alone. However, when phenotyping experiments are carried out in a rooting medium other than water or a nutrient solution, the medium itself has a polarization response that will affect the impedance measurements (e.g. [69]). While the interpretation of a mixed soil-root signal is more challenging, for sufficient root volume, the root polarization response is strong in comparison to soil [49], and recently, first studies have been conducted to better understand the effect of soil on the complex resistivity signature of root systems [69]. Disentangling the soil and root contributions to the polarization response will be needed to transfer the method to field phenotyping trials, and first steps have already been taken to apply sEIT to root systems at the field scale [89]. Therefore, to this date, in-situ field phenotyping with spectroscopic impedance methods is still challenging, although the method has strong potential to bridge the gap between laboratory and field phenotyping trials.

## Conclusions

In this study, we successfully used sEIT measurements to estimate the biomass and root surface area of four different crop root types in a water-filled rhizotron container. We utilized optimized electrode configurations for improved data acquisition and demonstrated their importance for the reconstruction of a polarization anomaly. The sEIT images reveal the insensitivity of the resistivity to the presence of roots, and contrarily, show the sensitivity of the frequency-dependent polarization signature to the presence of the root system. From the complex resistivity images, we derived integral polarization parameters for the root systems and established relationships between the polarization strength of the root zone and independently measured root traits. Different root architectures did not have an impact on the polarization strength, suggesting that in electrically low-contrast environments, the total polarization is mainly dependent on the presence of root matter and not on the orientation or type of the root segments. The recovered relaxation time values indicate that polarization takes place at the $\mu$m-scale, pointing at polarization processes within the root segments and not on the outer root surface. While the monocot plants share a slightly lower relaxation time in comparison to the dicots, no correlation with the root diameter was found. Although areas with low root biomass are prone to overestimation due to the smoothness constraint in the inversions, the overall correlation between estimated and validation root biomass is good. In future studies, research should be conducted towards the influence of root diameter and root cell size on the relaxation time obtained from sEIT measurements, as a link between these quantities could provide valuable additional information about the anatomy of a plant root system that is difficult to extract from the chargeability alone. Additionally, controlled experiments with soil as a rooting medium should be performed to advance sEIT towards a non-invasive phenotyping method at the field scale.

### Supplementary Information

**Additional file 1.**

## Data Availability

The datasets supporting the conclusions of this article are available in the Zenodo repository, 10.5281/zenodo.11085704.

## Appendix

see Figs. 12, 13, 14, 15.

## Abbreviations

BB: Black bean
DAS: Day after sowing
EDL: Electrical double layer
EIS: Electrical impedance spectroscopy
ERT: Electrical resistivity tomography
M: Maize
MALM: Mise-à-la-masse
PB: Pinto bean
PCC: Pearson correlation coefficient
RMS: Root-mean-square
SB: Soy bean
sEIT: Spectral electrical impedance tomography
SSIM: Structural similarity index
