# Supplementary material for: Quantitative phenotyping of crop roots with spectral electrical impedance tomography: a rhizotron study with optimized measurement design
Source: Plant Methods. 2024 Aug 2;20:118. doi: 10.1186/s13007-024-01247-7 (PMC11297745; doi:10.1186/s13007-024-01247-7)
Supplement: Supplementary file 1 — Additional file 1. [file 13007_2024_1247_MOESM1_ESM.pdf]

1 Raw data correction

Two corrections were performed on the recorded raw datasets: First, a correction to account for the 2D inversion of a dataset that was collected in a 3D domain, and secondly, a correction for polarization effects caused by the measurement setup.

Regarding the former, since the measurements are carried out in a 3D geometry and exhibit a 3D potential distribution, but forward modelling in the inversion is performed on a 2D grid, no variation of the potential within the depth of the rhizotron is falsely assumed. Therefore, the measured impedance magnitude values have to be corrected by a factor that is unique for each measurement configuration. These correction factors were computed with an auxiliary measurement that was performed on a water filled rhizotron without any roots, as described in [1]. Regarding the latter, two factors, namely the electrodes and used cables, cause unwanted influences on the measured polarization signal. Because the electrodes are made out of silver wire, the electrodes themselves polarize within the electric current path. While their size is too small to have a measurable influence on the impedance magnitude, they have a significant impact on the measured phase values through polarization processes at the electrode surface (e.g. [2]). To counteract this effect, previous studies used a technique to move the electrodes outside of the current path, for example as performed in [3] and [4]. However, this procedure was not possible with the used measurement setup because the wire electrodes were too thin to allow sufficient contact when not protruding into the rhizotron. In this study, instead, a background water measurement was performed prior to every plant measurement, capturing all unwanted polarization in the setup. The raw impedance phase spectra were then corrected for the background polarization according to

$$\varphi_{Z,\mathrm{corr}} = \varphi_{Z,\mathrm{meas}} - \varphi_{Z,\mathrm{background}}. \tag{1}$$

This approach does not only remove the influence of the polarized electrodes, but also inductive and capacitive effects of the measurement cables, as for example described in [5] or [6]. Note that in both of these works, more sophisticated correction methods using numerical modelling and additional corrective measurements were employed. However, since the measurements in this study were performed solely for one timestep

in water and therefore did not have any other varying contributions to the polarization signal, we deemed the approach used here as sufficient and more practicable. The correction by subtraction is only feasible as long as the cable location stays constant, so we took care to not move cables between correction and actual plant measurements.

References

[1] Weigand M, Kemna A. Multi-frequency electrical impedance tomography as a non-invasive tool to characterize and monitor crop root systems. Biogeosciences. 2017;14(4):921–939. https://doi.org/10.5194/bg-14-921-2017.

[2] Zimmermann E, Huisman JA. The effect of heterogeneous contact impedances on complex resistivity measurements. Geophysical Journal International. 2024;236(3):1234–1245. https://doi.org/10.1093/gji/ggad477.

[3] Weigand M, Kemna A. Imaging and functional characterization of crop root systems using spectroscopic electrical impedance measurements. Plant and Soil. 2019;435(1-2):201–224. https://doi.org/10.1007/s11104-018-3867-3.

[4] Tsukanov K, Schwartz N. Relationship between wheat root properties and its electrical signature using the spectral induced polarization method. Vadose Zone Journal. 2020;19(1):e20014. https://doi.org/10.1002/vzj2.20014.

[5] Zhao Y, Zimmermann E, Huisman JA, Treichel A, Wolters B, van Waasen S, et al. Broadband EIT borehole measurements with high phase accuracy using numerical corrections of electromagnetic coupling effects. Measurement Science and Technology. 2013;24(8):085005. https://doi.org/10.1088/0957-0233/24/8/085005.

[6] Weigand M, Zimmermann E, Michels V, Huisman JA, Kemna A. Design and operation of a long-term monitoring system for spectral electrical impedance tomography (sEIT). Geoscientific Instrumentation, Methods and Data Systems. 2022;11(2):413–433. https://doi.org/10.5194/gi-11-413-2022.
